# Polyphenols as Therapeutics in Respiratory Diseases: Moving from Preclinical Evidence to Potential Clinical Applications

**DOI:** 10.7150/ijbs.93875

**Published:** 2024-06-03

**Authors:** Talha Bin Emran, Taslima Akter Eva, Mehrukh Zehravi, Fahadul Islam, Jishan Khan, Shaik Kareemulla, Uppuluri Varuna Naga Venkata Arjun, Anitha Balakrishnan, Poonam Popatrao Taru, Firzan Nainu, Emil Salim, Safia Obaidur Rab, Mohamed H. Nafady, Polrat Wilairatana, Moon Nyeo Park, Bonglee Kim

**Affiliations:** 1 Department of Pathology and Laboratory Medicine, Warren Alpert Medical School, Brown University, Providence, RI 02912, USA.; 2 Legorreta Cancer Center, Brown University, Providence, RI 02912, USA.; 3 Department of Pharmacy, Faculty of Allied Health Sciences, Daffodil International University, Dhaka 1207, Bangladesh.; 4 Department of Pharmacy, Faculty of Biological Sciences, University of Chittagong, Chittagong 4331, Bangladesh.; 5 Department of Clinical Pharmacy, College of Dentistry & Pharmacy, Buraydah Private Colleges, Buraydah 51418, Saudi Arabia.; 6 Department of Pharmacy, International Islamic University Chittagong, Chittagong 4318, Bangladesh.; 7 Department of Pharmacy Practice, M. M. College of Pharmacy (Maharishi Markandeshwar Deemed University), Mullana-Ambala, Haryana 133207, India.; 8 Department of Pharmaceutics, School of Pharmaceutical Sciences, Vels Institute of Science, Technology, and Advanced Studies (VISTAS), Tamil Nadu, India.; 9 Department of Pharmaceutics, GRT Institute of Pharmaceutical Education and Research, Tiruttani, India.; 10 Department of Pharmacognosy, School of Pharmacy, Vishwakarma University, Kondhwa, Pune, India.; 11 Department of Pharmacy, Faculty of Pharmacy, Hasanuddin University, Makassar 90245, Indonesia.; 12 Department of Pharmacology and Clinical/Community Pharmacy, Faculty of Pharmacy, Universitas Sumatera Utara, Medan, 20155, Indonesia.; 13 Department of Clinical Laboratory Sciences, College of Applied Medical Sciences, King Khalid University, Abha, Saudi Arabia.; 14 Faculty of Applied Health Science Technology, Misr University for Science and Technology, Giza 12568, Egypt.; 15 Department of Clinical Tropical Medicine, Faculty of Tropical Medicine, Mahidol University, Bangkok 10400, Thailand.; 16 Department of Pathology, College of Korean Medicine, Kyung Hee University, 26 Kyungheedae-ro, Dongdaemun-gu, Seoul, 02453, Republic of Korea.

**Keywords:** Polyphenols, Respiratory diseases, Lung cancer, Asthma, ARDS, Tuberculosis

## Abstract

Respiratory diseases are the most common and severe health complication and a leading cause of death worldwide. Despite breakthroughs in diagnosis and treatment, few safe and effective therapeutics have been reported. Phytochemicals are gaining popularity due to their beneficial effects and low toxicity. Polyphenols are secondary metabolites with high molecular weights found at high levels in natural food sources such as fruits, vegetables, grains, and citrus seeds. Over recent decades, polyphenols and their beneficial effects on human health have been the subject of intense research, with notable successes in preventing major chronic non-communicable diseases. Many respiratory syndromes can be treated effectively with polyphenolic supplements, including acute lung damage, pulmonary fibrosis, asthma, pulmonary hypertension, and lung cancer. This review summarizes the role of polyphenols in respiratory conditions with sufficient experimental data, highlights polyphenols with beneficial effects for each, and identifies those with therapeutic potential and their underlying mechanisms. Moreover, clinical studies and future research opportunities in this area are discussed.

## Introduction

Respiratory or lung diseases are medical conditions that impede the gas exchange process in air-breathing animals by affecting the lungs and other respiratory tissues. In addition to the lungs, these conditions also affect other respiratory organs, including the trachea, bronchi, bronchioles, alveoli, pleurae, pleural cavity, and respiratory nerves and muscles 1. Asthma, characterized by recurrent episodes of wheezing, coughing, and breathlessness, arises from chronic airway inflammation and hyperresponsiveness. Triggered by allergens, irritants, or even emotional stress, inflammatory cells and mediators including histamine infiltrate the airways, causing their narrowing and mucus overproduction. This “bronchoconstriction” hinders airflow, leading to the characteristic symptoms 2. COPD, encompassing emphysema and chronic bronchitis, is a progressive, often irreversible disease. While cigarette smoking is the leading culprit, occupational exposure and genetics also play a role 3. Emphysema involves the destruction of the air sacs (alveoli), diminishing the surface area for gas exchange. Chronic bronchitis, on the other hand, features chronic inflammation and mucus hypersecretion, obstructing the airways. Both mechanisms culminate in breathlessness, reduced exercise tolerance, and impaired quality of life 4. According to GOLD report 2023, the global prevalence of COPD oscillates around 12% of the general population. The incidence of COPD is expected to rise over the next 40 years. By 2060, it is estimated that there may be over 5.4 million deaths annually from COPD 5.

Polyphenols are phytochemical compounds present in fruits, vegetables, and grains. These chemicals provide several health advantages, including immune-modulating, vasodilating, and antioxidant properties 6. Natural food sources contain the most common bioactive molecules, particularly polyphenols that comprise two or more phenolic rings with attached hydroxyl groups in their structures. Polyphenolic compounds include flavonoids, flavanones, and anthocyanins, which are present at high concentrations in various fruits and vegetables. Their pharmacological and therapeutic profiles have been studied extensively, particularly in the context of respiratory diseases 7. For example, the study of 582 Hawaiian lung cancer patients found a significant inverse relationship between lung cancer and the polyphenols quercetin and naringin, with a 40-50% lower risk of lung cancer in patients who consumed the most polyphenols compared to those who consumed the least 8. In addition, other studies have reported favorable findings with polyphenols in lung cancer 9. Resveratrol exerts its potential health benefits through various pathways and cellular components. It acts primarily as an antioxidant, scavenging harmful free radicals and mitigating oxidative stress, which is linked to multiple diseases.

Additionally, it influences several cellular signaling pathways, including those involving SIRT1, AMPK, and Nrf2. These pathways are involved in regulating cellular metabolism, inflammation, and stress responses 10. Polyphenols exhibit several health advantages, including hypoglycemic, anti-inflammatory, and cancer-preventive properties. Additionally, they play a significant role in improving the flavor of food 11. The exploration of non-toxic and cost-effective polyphenols, such as epigallocatechin 3-gallate and myricetin, for health improvement and disease treatment has recently attracted substantial research attention. The recent COVID-19 pandemic has provided a unique opportunity for the investigation and identification of polyphenols capable of treating viral infections, as well as gathering the evidence needed to address the significant challenges presented by public health emergencies. Polyphenols hold great potential as a starting point for further drug development for the treatment and prevention of SARS-CoV-2 infection and diseases associated with the respiratory system owing to their excellent safety, broad-spectrum antiviral activities, and multi-organ protective capacity 12,13.

This review provides a comprehensive summary of the pharmacotherapeutic properties of dietary polyphenols in respiratory diseases and identifies promising candidates for novel drug discovery.

## Polyphenols: An overview

**Figure 1:**
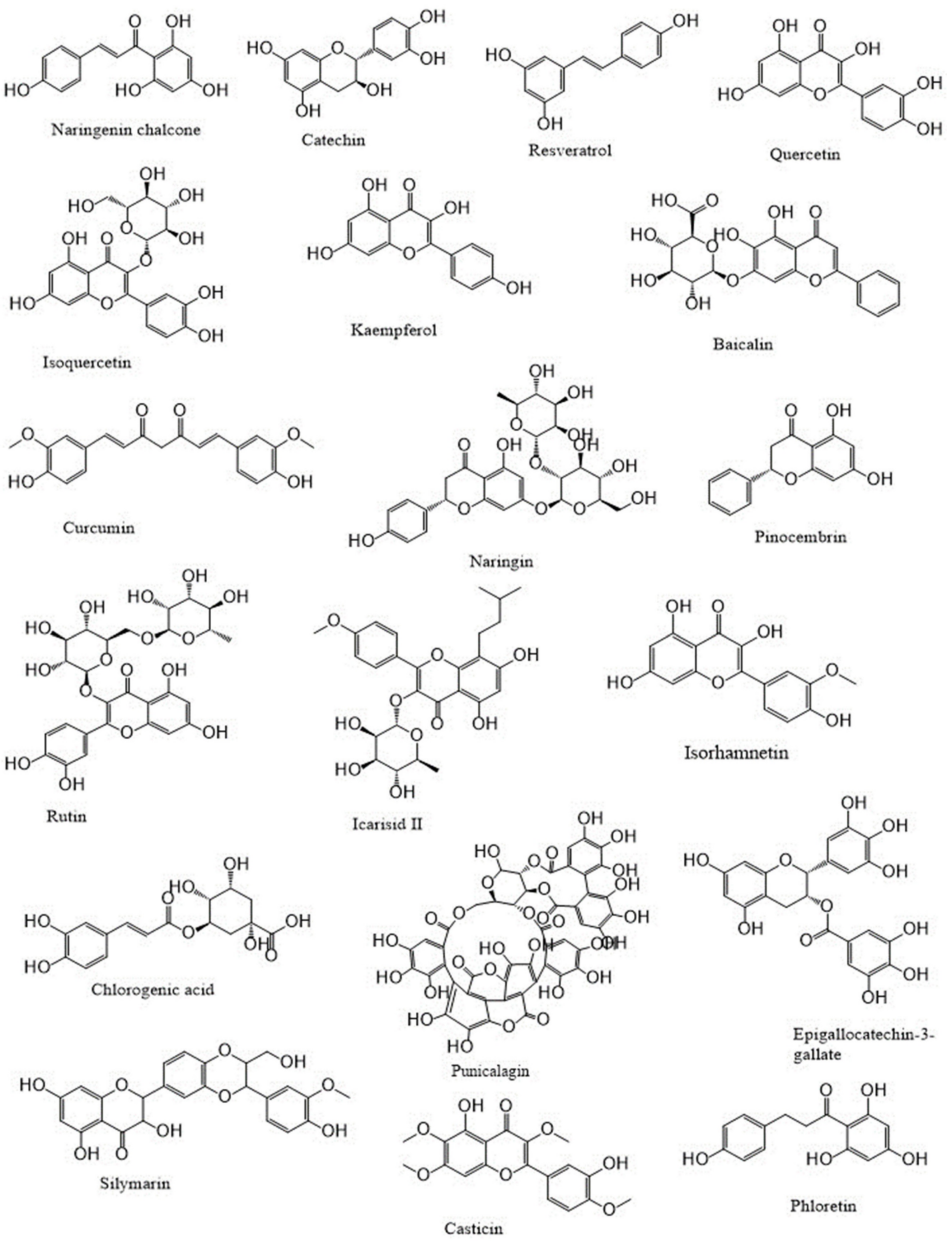

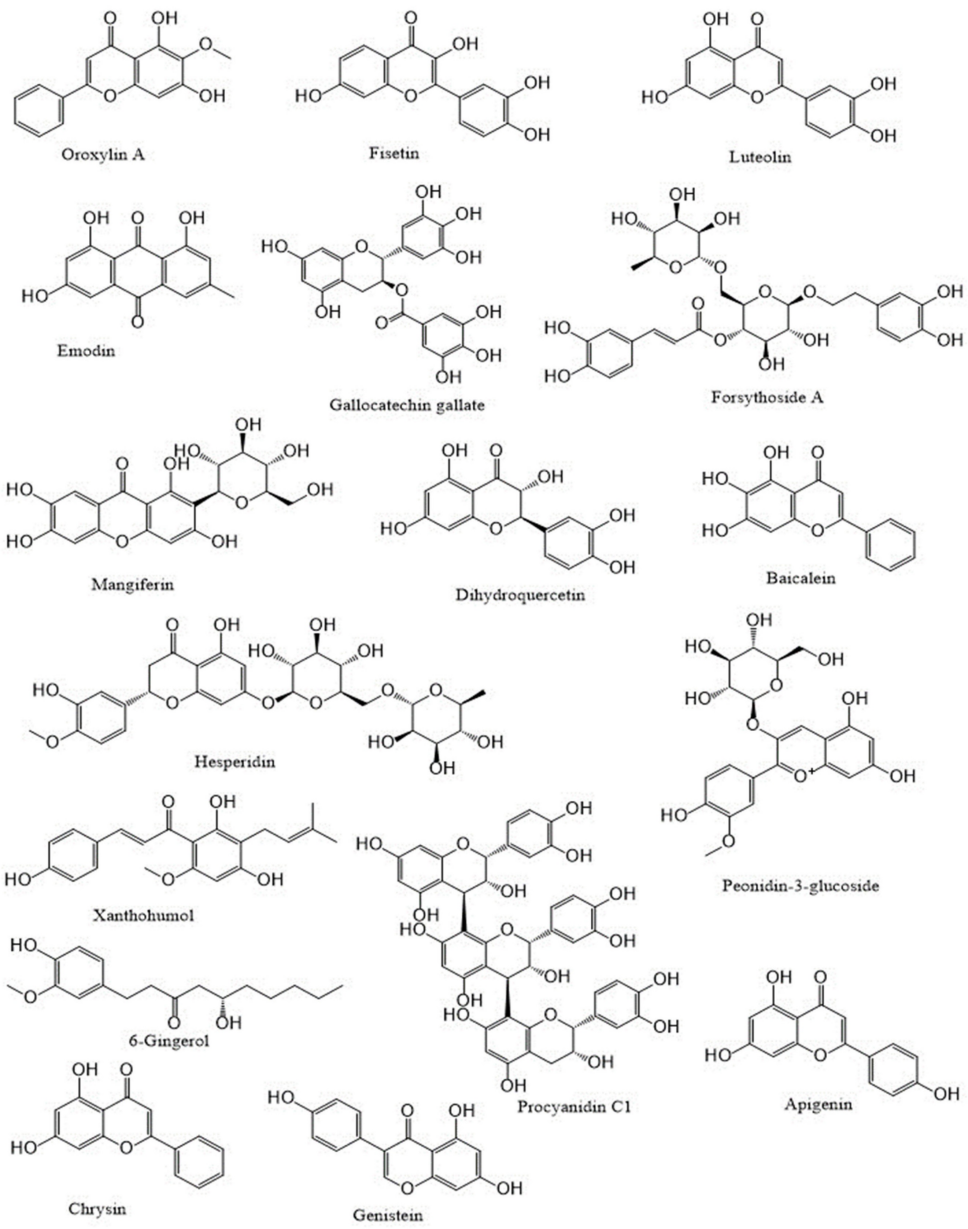
Polyphenols as therapeutics in respiratory diseases.

Polyphenols are a category of bioactive chemicals found in plant-based diets 14,15. Polyphenols are small organic compounds that have an aromatic ring, such as benzene or phenol, with one or more hydroxyl groups in their structure 16,17. Polyphenols generated from foods are secondary compounds often present in fruits and vegetables and provide many health advantages to humans 18,19. Polyphenols are a diverse set of molecules that consist of phenolic acids, flavonoids (such as flavonols, flavanones, flavan-3-ols, flavones, anthocyanins, and isoflavones), lignans, stilbenes and, in some classifications, tannins and coumarins 20-22. Flavonoids are a primary category of dietary polyphenols known for their potent antioxidant and anti-carcinogenic effects 23. Flavonoids are present in fruits, vegetables, nuts, seeds, coffee, wine, and tea, and they have notable antioxidant properties linked to conditions including cancer, atherosclerosis, and Alzheimer's disease 24-26. They are categorized based on their chemical composition, level of unsaturation, and carbon ring oxidation. Flavonoids are divided into many subgroups, including anthoxanthins (flavanone and flavanol), flavanones, flavanonols, flavans, chalchones, anthocyanidins, and isoflavonoids 27,28. Flavonoids consist of a fundamental 15-carbon flavone structure, C6-C3-C6, with two benzene rings (A and B) connected by a three-carbon pyran ring (C). The location of the catechol B-ring on the pyran C-ring and the quantity and placement of hydroxy groups on the catechol group of the B-ring impact the antioxidant potential of flavonoids 29,30. Flavonols are similar in structure to flavones and are distinguished by a hydroxyl group (-OH) at the C3 position and a carbonyl function (C=O) at the C4 position on the C ring 31,32. Quercetin and kaempferol are distinguished by the presence of an extra hydroxyl group at the R1 position in the quercetin molecule 33,34. Flavonols, particularly quercetin, exhibit a wide variety of biological roles. Due to their impact on cell-signaling pathways related to oxidative stress and inflammation, they may enhance lipid metabolism, vascular function, blood pressure, and glucose metabolism 26,35,36. The flavanone family is prevalent in fruits and fruit juices of the Citrus species, making up around 95% of flavonoids in this subclass 37,38. The only chemical structural distinction between flavanones and other flavonoids is the unsaturated double bond located between positions C2′ and C3′ of the C-ring 39,40. Neohesperidin, hesperidin, and hesperetin (**Figure 1**) are citrus flavonoids belonging to the flavanones subclass known for their anti-inflammatory and antioxidant properties 41.

Polyphenols, a diverse group of plant-based compounds, can be broadly categorized based on their mechanisms of action against respiratory diseases 42. Some, like flavonoids and curcumin, act as antioxidants, scavenging harmful free radicals that contribute to lung inflammation 43. Others, like resveratrol, possess anti-inflammatory properties, dampening the immune response that can worsen respiratory conditions like asthma 44. Additionally, certain polyphenols, such as quercetin, exhibit bronchodilatory effects, relaxing airway muscles and easing breathing difficulties 45. It's important to note that research on the effectiveness of individual polyphenols and their optimal dosages for respiratory ailments is ongoing, and seeking professional medical advice is crucial before using any supplements 46.

## Role of polyphenols in respiratory diseases

### COPD

An estimated 300 million new cases of COPD are diagnosed annually worldwide 47. Individuals suffering from bronchitis, characterized by shortness of breath, are at risk of developing long-term respiratory problems if it is not effectively treated. Shortness of breath is one of the most common symptoms of long-term respiratory issues 48. Over the last decade, there has been a growing emphasis on the creation of efficient methods and tools for the early identification of COPD 49. Inflammation caused by COPD has been associated with the risk of lung cancer. While there is currently no effective treatment available for COPD 50, there is evidence that flavonoids may be beneficial in treating this disease 51,52. The protective functions of quercetin against chronic lung obstruction and pulmonary emphysema progression have been investigated in animal models 53.

**Figure 2:**
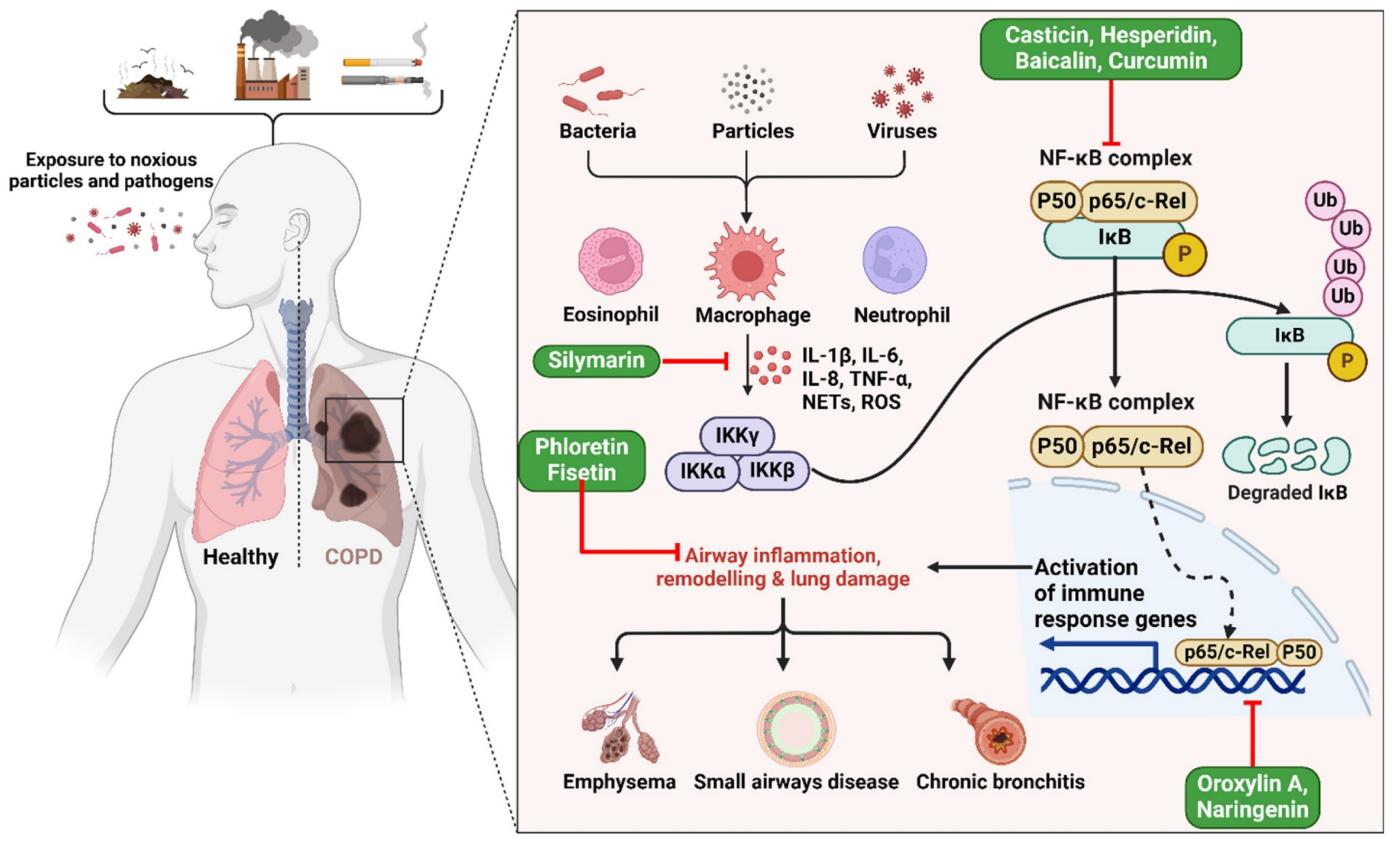
Illustration representing the probable site of action of bioactive polyphenols. Upon exposure to harmful particles and pathogens, macrophages are activated, triggering the release of pro-inflammatory cytokines (e.g., IL-6, TNF-α) and reactive oxygen species. This cascade activates the NF-κB pathway and immune response genes, resulting in airway inflammation, structural remodeling, and lung damage, ultimately culminating in COPD. The figure was designed by Biorender.com program (https://biorender.com/, accessed on 20 March 2024).

Studies have displayed that baicalin has a notable capacity to enhance COPD and inflammation in animal models and cell cultures 54. Baicalin was administered to six groups in a rat lung cancer model, both with and without exposure to cigarette smoke (CS) 55. An optical microscope was used to assess the leukocyte count in the bronchoalveolar lavage fluid (BALF). The research revealed that baicalin effectively mitigated inflammation caused by CS. The researchers used CS extract (CSE) to stimulate type II pneumocytes and then examined the impacts of both CSE and pyrrolidine dithiocarbamate (PDTC). The study demonstrates that baicalin has noteworthy anti-inflammatory properties in rat models of COPD caused by CS, as well as in cell models created by CSE. Furthermore, the efficacy of baicalin rises proportionally with higher dosages 55. Another study found casticin to protect the lungs against COPD by blocking NF-κB, improving pulmonary performance, and lowering oxidative stress and inflammation throughout the body (**Figure 2**) 50. When rats were exposed to CSE at doses of 10, 20, and 30 mg/kg, plasma levels of leptin, C-reactive protein, and pro-inflammatory cytokines interleukin 1 (IL-1), IL-6, and TNF-α were restored to near normal levels by casticin (CST) treatment 50. Activating the nuclear factor E2-related factor 2 (Nrf2) signaling pathway with oroxylin A was found to reduce oxidative stress and inflammation in the lungs, which may represent an effective preventative strategy against CS-induced lung inflammation and COPD 56. In addition, studies exploring the effects of oroxylin A on RAW264.7 and BEAS-2B bronchial epithelial found it attenuated CS-induced cytokine production and 3-nitrotyrosine and 8-isoprostane levels, as well as histological abnormalities in the lungs of mice given daily intraperitoneal injections of oroxylin A for five days before CS treatment. However, oroxylin A increases glutathione (GSH) levels and glucocorticoid receptor (GR) activity in lung tissues. Moreover, cells treated with oroxylin A and then exposed to CSE showed substantial increases in NRF2 and GSH levels. Furthermore, oroxylin A increases Nrf2 binding to antioxidant response elements (AREs), increasing the expression of genes involved in the antioxidant response, including heme oxygenase 1 (*HO-1*), glutathione peroxidase (*GPx*), and GR, in CSE-stimulated cells 56.

Fisetin has been shown to be an effective drug for treating inflammation-related lung illnesses such as COPD 57, reducing NF-κB binding in the IL-8 promoter region in NCI-H292 lung epithelial cells, resulting in reduced IL-8 production in response to TNF-α 57. The suppression of NF-κB signaling by fisetin is most likely due to the disruption of its upstream regulators, including IκB kinase (IKK) and IB. Indeed, fisetin may have an inhibitory effect on these regulators, reducing NF-κB activation and IL-8 synthesis 57. A case-control COPD study aimed to evaluate the effects of resveratrol and genistein on NF-κB, TNF-α, and matrix metallopeptidase 9 (MMP9) expression. The study involved 34 COPD patients and 30 healthy individuals in four groups: untreated control, dexamethasone-treated, resveratrol-treated (12.5 mol/L), and genistein-treated (25 mol/L). NF-κB-positive cell numbers increased with resveratrol concentration, while genistein-positive cell numbers decreased with lower dosages. TNF-α levels in resveratrol and genistein-treated groups correlated positively with the percentage of NF-κB-positive cells. Increased resveratrol concentrations were associated with lower MMP9 levels in a dose-dependent manner. Phytonutrient levels stabilized and increased slightly with resveratrol concentrations. MMP9 levels were inversely correlated with genistein concentration. Resveratrol was found most effective at 12.5 mol/L, while genistein was most effective at 25 µmol/L 58.

### Asthma

**Figure 3:**
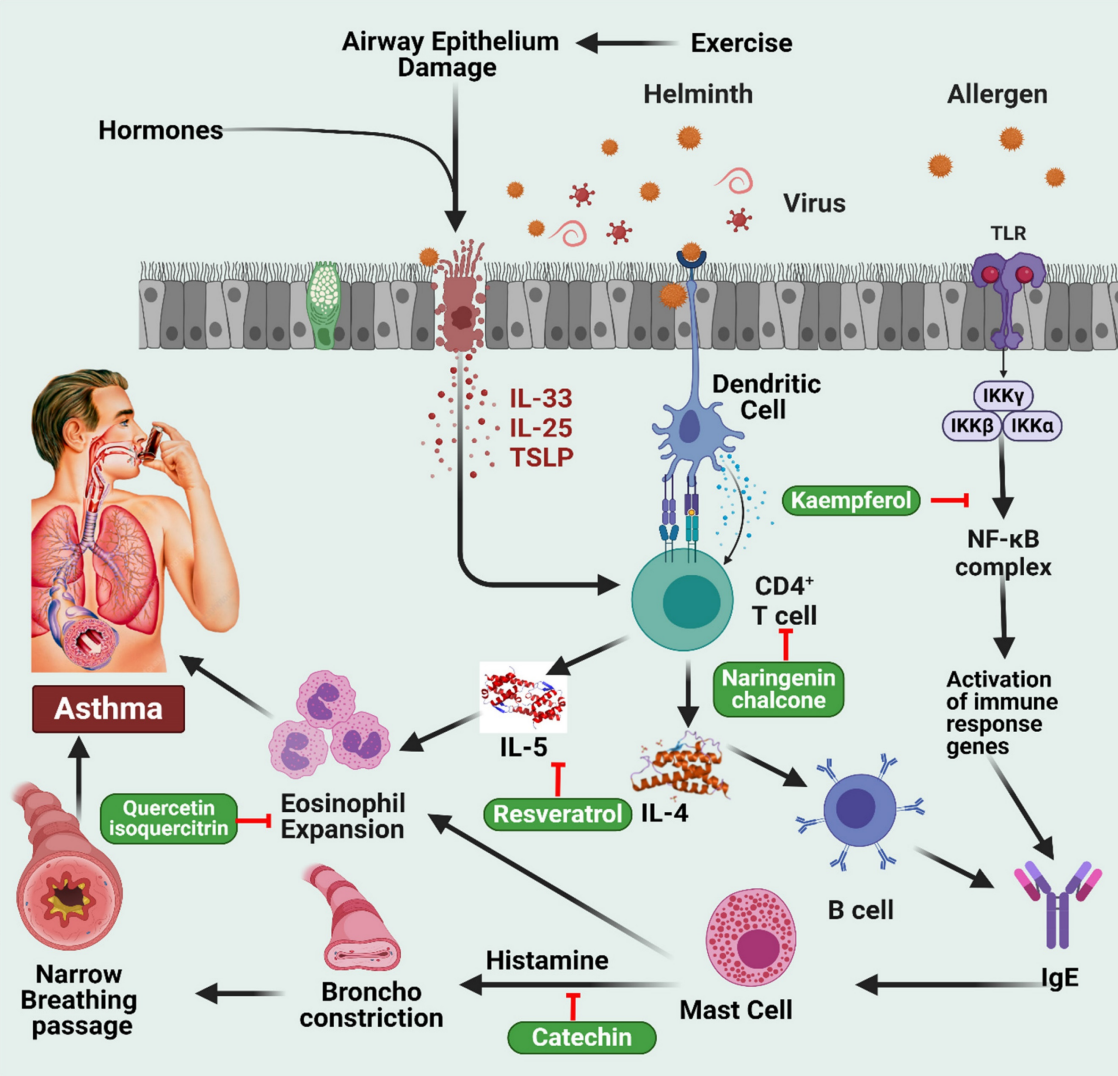
Illustration representing the sites of action of different polyphenols in asthma pathway. Hormonal fluctuations and airway epithelium damage trigger the secretion of IL-33, IL-25, and TSLP, which, in collaboration with dendritic cells, activate CD4+ T cells. This sequence leads to B cell stimulation of IgE production, activating mast cells and causing eosinophil expansion, resulting in asthma. The involvement of the NF-κB complex activates immune response genes, stimulating IgE and histamine release from mast cells. Histamine induces bronchoconstriction, contributing to asthma-related respiratory distress. The figure was designed by Biorender.com program (https://biorender.com/, accessed on 20 March 2024).

Flavonoids have been found to decrease airway inflammation and the immunoglobulin E (IgE) response in asthmatic animals, dependent on their *in vitro* anti-asthmatic properties. Apigenin, fisetin, and luteolin are potent inhibitors of interleukin-4 (IL-4) production (**Figure 3**) 59,60. Ovalbumin (OVA) was injected intraperitoneally into BALB/c mice on days 0, 7, and 14 to sensitize them. They then inhaled OVA daily by aerosol on days 19 to 23. Daily oral administration of luteolin at 0.1, 1.0, or 10 mg/kg was provided throughout the sensitization period. After sensitization, oral administration of luteolin at 1 mg/kg was provided on days 26 to 32. Luteolin was found to have a considerable suppressive effect on OVA-induced bronchial hyper-reactivity and airway bronchoconstriction at the dosages investigated, both during and after sensitization 61. In addition, Luteolin treatment reduced OVA-specific IgE serum levels and increased IFN-γ but decreased IL-4 and interleukin 5 (IL-5) levels in BALF. Moreover, a preventive effect of luteolin and omega-3 polyunsaturated fatty acids (PUFA) supplementation on airway responsiveness was observed in cats exposed to *Ascaris suum* 62. Studies have injected mice intraperitoneally with apigenin at doses of 5 and 10 mg/kg before the final OVA challenge to explore whether it can reduce asthmatic symptoms in an OVA-sensitized mouse model. Apigenin treatment prevented mice from developing asthmatic symptoms such as elevated serum IgE levels, eosinophil accumulation, and IL-4, IL-5, and erythropoietin (EPO) activity in blood and lung fluid 63. Similar effects were also observed at apigenin doses of 2 and 20 mg/kg treatment in an OVA-induced asthmatic model 64. Intraperitoneal injections of fisetin at 3 mg/kg were found to reduce hyperplasia, inflammation, and hyperresponsiveness of the airways in mice exposed to OVA aerosols 65. In addition, this treatment decreased the expression of the primary initiators of allergic airway inflammation and T helper 2 (Th2)-associated cytokines IL-4, IL-5, and interleukin 13 (IL-13) in lung tissues. While it had been previously reported that fisetin inhibits NF-κB activity 66, it also impairs NF-κB activation in OVA-induced lung tissues. Moreover, fisetin suppressed OVA-induced increases in eosinophil count, total cell count, and IL-4, IL-5, and IL-13 levels in BALF in a dose-dependent manner when administered intravenously at 0.3, 1, or 3 mg/kg before OVA aerosol challenge on days 22 to 24 67. Finally, it also reduced the effects of OVA on lung tissue eosinophilia, airway mucus buildup, airway hyperresponsiveness, and expression of adhesion molecules, chitinase (*CHIA*), interleukins 17 (*IL-17*) and 33 (*IL-33*), major airway glycoprotein mucin 5AC oligomeric mucus/gel-forming (*Muc5ac*), and inducible nitric oxide synthase (*iNOS*).

### Lung cancer

It is estimated that 2 million individuals are diagnosed with lung cancer annually, and 1.8 million individuals die from it 68, making it the leading cause of cancer-related death worldwide 69. Small-cell lung cancer (SCLC) and non-small-cell lung cancer (NSCLC) are two lung cancer subtypes distinguished by their tumor-causing cells. Squamous cell carcinoma (SCC), adenocarcinomas (cancer of glandular cells), and neuroendocrine tumors such as SCLC, large cell neuroendocrine carcinoma (LCNEC), and carcinoids are the most common based on recent reports 70,71. Polyphenols found abundantly in fruits, vegetables, and beverages like tea hold promise in inhibiting lung cancer development through various mechanisms. They act as antioxidants, scavenging free radicals that damage DNA and contribute to cancer prevention 72. Additionally, they can block the activation of pro-carcinogens by enzymes, preventing their harmful effects. Polyphenols also interfere with cancer cell signaling pathways, hindering their uncontrolled growth and promoting cell death (apoptosis) 73.

**Figure 4:**
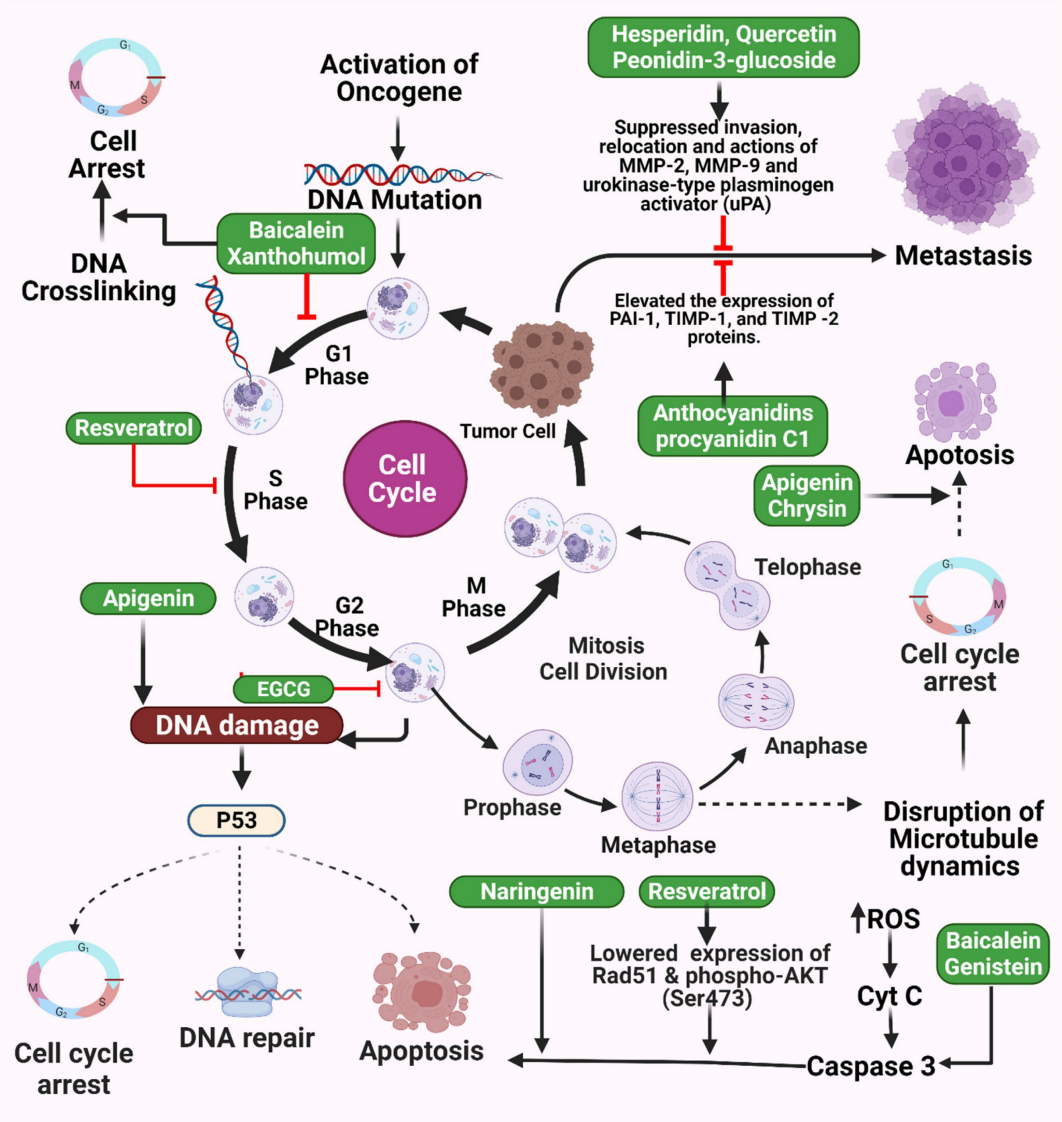
Illustration representing the sites of action of different polyphenols in cancer pathway. Activation of oncogene triggers abnormal cell division and finally results in tumor cells followed by metastasis. DNA crosslinking, DNA damage, abnormal p53 activities, and microtubule disruption collectively result in abnormal apoptosis and cell cycle arrest which cause cancer. Different polyphenols probably work on these pathways to prevent cancer. The figure was designed by Biorender.com program (https://biorender.com/, accessed on 20 March 2024).

Antioxidants such as flavonoids and proanthocyanidins found in high concentrations in fruits and vegetables can help reduce lung cancer risk. Nutrient polyphenols are involved in regulating cell survival pathways, which, in addition to their antioxidant properties, contribute to their anticancer and antimutagenic effects. There is increasing data from *in vitro*, *in vivo*, and epidemiological studies supporting the chemopreventive influence of polyphenols and their role in cancer prevention 74. Despite recent advances in therapy, less than one-fifth of lung cancer patients live beyond five years 75. According to a recent study, resveratrol inhibits NSCLC cell growth by causing early necrosis through reactive oxygen species (ROS)-induced DNA damage. Resveratrol may increase ROS production in A549 and H460 lung cancer cells by regulating nicotinamide adenine dinucleotide phosphate (NADPH) oxidase 5 (*Nox5*) expression in cells 76. However, trans-resveratrol induces apoptosis in human adenocarcinoma epithelial cells via mitochondrial-dependent pathways 77. Resveratrol has also been found to have anti-apoptotic and anti-proliferative effects on lung cancer cells. Resveratrol has been found to bind to synthetic or natural promoters of early growth response 1 (Egr-1) and to enhance the expression of growth arrest and DNA damage-inducible (*GADD45*) in A549 lung cancer cells (**Figure 4**). Egr-1 mRNA and protein levels were elevated in A549 lung cancer cells grown with 100 M resveratrol within two hours of administration and increased in a dose-dependent manner when the resveratrol was administered for six hours at concentrations of 0, 25, 50, and 100 µM 78.

While studies of lung carcinogens are limited, those on phytochemicals are becoming more common. Chromium-induced caspase-3 (CASP3) activation, ROS production, and protein crosslinking in DNA decreased in BEAS-2B cells treated with epigallocatechin gallate (EGCG) in a dose-dependent manner 79. Curcuminoid bisdemethoxycurcumin derived from the turmeric plant *Curcuma longa* has been shown to prevent premature aging of normal lung fibroblast WI-38 cells, potentially through the sirtuin 1 (Sirt1)/AMP-activated protein kinase (AMPK) signaling pathway 80. Dieckol was found to reduce the invasive and apoptotic abilities of A549 lung cancer cells *in vitro* by inhibiting signaling through phosphoinositide 3-kinase (PI3K), protein kinase B (AKT1), and mammalian target of rapamycin (mTOR) and activation of the tumor suppressor protein E cadherin (CDH1) 81. Therefore, dieckol may represent a natural anticancer drug effective against NSCLC 81,82. In addition, kaempferol was found to inhibit Akt1-mediated phosphorylation of Thr179 in Smad3, reducing the epithelial-to-mesenchymal transition (EMT) induced by transforming growth factor β 1 (TGF-β1) in lung cancer cell transplants 83. This was the first demonstration of a potential molecular mechanism for kaempferol's anti-cancer activity that might also mediate camphor's suppression of malignant cell proliferation. Moreover, it showed that phosphorylation of the Smad3 linker region is required for EMT and cell migration and induced by TGF-β1, with lower EMT and cell migration in the presence of kaempferol due to low levels of Thr179 Smad3 phosphorylation. In contrast, phosphorylation of Smad3 did not occur at Ser204, Ser208, or Ser213. Moreover, Akt1 is essential for cell transplantation as well as EMT formation induced by TGF-β1. In addition, it has been shown that Akt1 can directly phosphorylate Smad3 at Thr179, which ultimately inhibits Akt1 phosphorylation induced by TGF-1 with camphor 82.

### TB

Tuberculosis (TB) is a leading cause of death caused by the bacteria *Mycobacterium tuberculosis*. According to recent data, there were 10.4 million new TB infections in the United States in 2017 84, of which 64% were male, and most were over 15 years old. Co-infection with the human immunodeficiency virus (HIV) is found in 1 in 10 TB sufferers, 72% of whom live in Africa. Isoniazid can cause neuritis, but other side effects, such as ethambutol and rifampin, can also occur. Therefore, there is an urgent need for new TB drugs with minimal side effects, even when used in combination 85.

Epigallocatechin gallate, a green tea catechin with antimicrobial properties, and its derivatives have been studied for their inhibitory effects on Mycobacterium smegmatis. Enoyl reductase (Inh A), a drug target inhibited by isoniazid, was docked with the geometrically optimized conformation of epigallocatechin gallate and two of its derivatives. The Ames test was performed to assess the mutagenic potential of epigallocatechin gallate. Inhibin subunit alpha (InhA) has been successfully docked with a docking capacity of -9.38 kcal mol^-1^. The minimum inhibitory concentrations for epigallocatechin gallate, per-propionate, and permethyl were 128 (58.2% inhibition), 8 (32.9%), and 4 (12.5%) µg/ml, respectively. Epigallocatechin gallate showed a cytotoxicity of 18.6% at eight µg/ml, while the two derivatives did not show any toxicity. The non-mutagenic epigallocatechin gallate inhibits *M. smegmatis* growth and warrants further investigation as an adjunct therapy for pathogenic mycobacteria 86.

**Figure 5:**
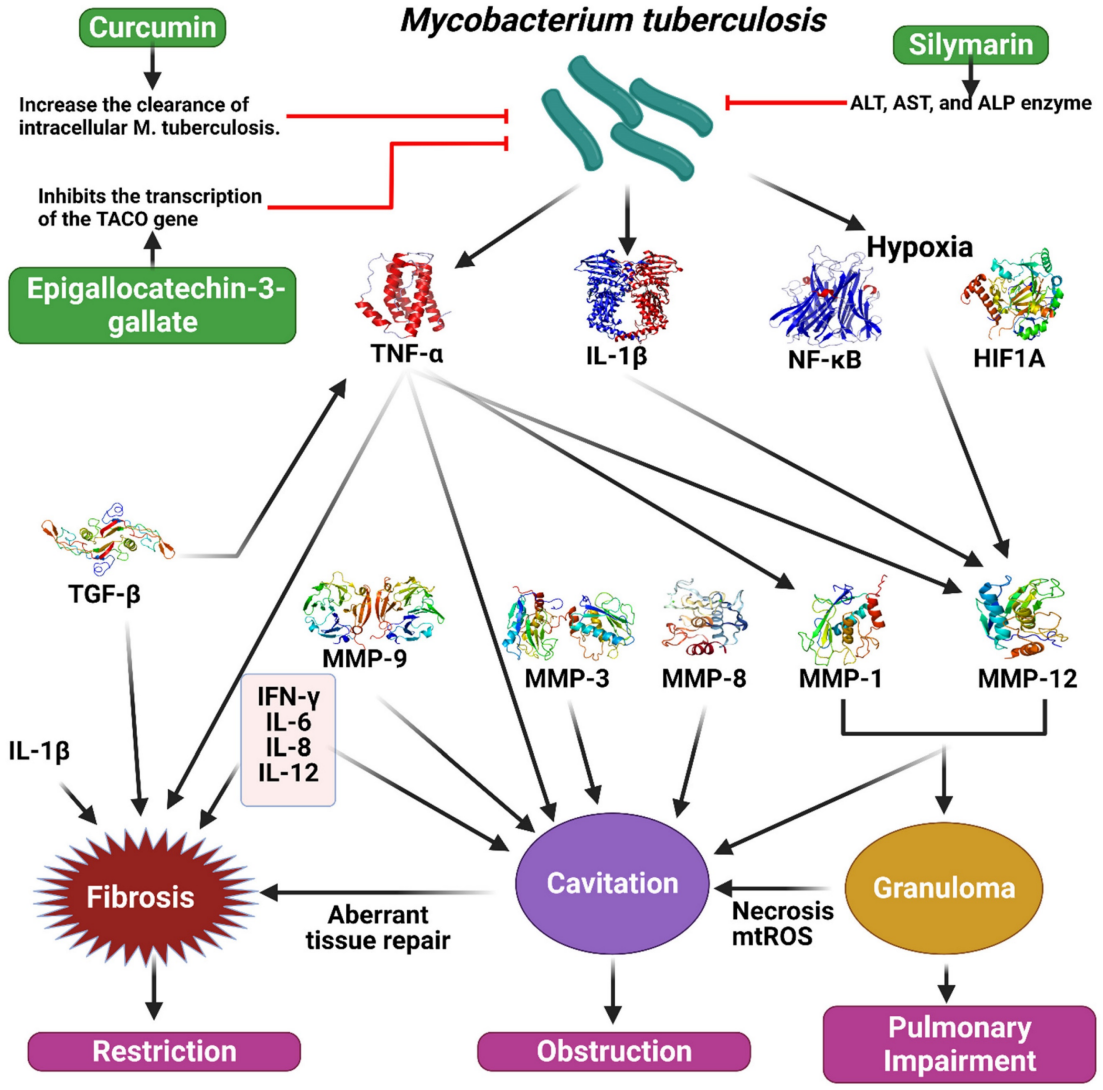
Illustration representing the sites of action of different polyphenols in tuberculosis pathway. *Mycobacterium tuberculosis* stimulate the secretion of TNF-alpha, IL-1B, NF-kB, HIF1A, which cause the activation of different MMPs and TGF-B. As a result, fibrosis, granuloma, and cavitation occur which ultimately create restriction, obstruction and pulmonary impairment in tuberculosis. Polyphenols could work on this pathway to prevent tuberculosis. The figure was designed by Biorender.com program (https://biorender.com/, accessed on 20 March 2024).

According to a recent study, TB may alter the effect of curcumin, the primary active ingredient in turmeric, on intracellular clearance. They found curcumin reduces the intracellular concentration of *M. tuberculosis* in THP-1 cells by 10, 30, or 50 mg/mL (11, 47, and 67%, respectively) after infection. In addition, there was a statistically significant increase in the apoptotic rate of THP-1 cells after treatment with curcumin. In addition, LC3-I and LC3-II autophagy markers have been shown to be strongly induced by curcumin during TB infection. Moreover, intracellular TB load was reduced by 73% in primary human alveolar macrophages when curcumin was given four days after infection (**Figure 5**) 87.

### Acute lung injury (ALI) and acute respiratory distress syndrome (ARDS)

Septic shock, trauma, pneumonia, and frequent blood transfusions are among the conditions that lead to death in patients with pancreatitis, multiple organ failure, lung injury, and acute respiratory syndrome (ARDS), respectively 88. Acute lung injury (ALI) characterized by inflammation and blood clot formation, is often seen as a consequence of SARS-COV-2 infection. Particularly in intensive care, ARDS is a significant problem and can be triggered by ventilation with too high pressure 89. Proinflammatory cytokines, including TNF-α, IL-1, and IL-6, are critical components of ALI/ARDS due to rapid lung tissue loss, neutrophil infiltration, and parenchymal inflammatory reactions 90. Despite advances in ALI/ ARDS pathobiology over recent decades, the significant mortality rate (approximately 40%) associated with ALI/ARDS is poorly understood 88,91. The incidence of severe ARDS had a significant surge on a global scale during the COVID-19 epidemic, resulting in a substantial fatality rate 92. ARDS is still recoverable in the absence of treatment. Individuals are often treated with N-acetylcysteine (NAC) and other antioxidants, such as low tide breathing and water restriction. However, their survival rates did not improve as a result of these therapeutic approaches 93. Studies on rats have shown that the polyphenolic compound curcumin regulates IL-10 immunomodulation against lung injury. In the cecal ligation and puncture (CLP) mouse model, lung damage was improved with 20 mg/ml of curcumin administered intraperitoneally 94. Curcumin can reduce ALI severity and uncontrolled inflammation by promoting the differentiation of naïve CD4+ T cells to CD4+ CD25+ FOXP3+ Tregs and convert macrophages from M1 to M2, potentially influencing IL-10 immune modulation through Treg differentiation 94.

Flavonoid compounds have been shown to reduce the acute lung damage caused by lipopolysaccharide (LPS). Chen *et al.* showed that 100 mg/kg kaempferol administered intragastrically to male BALB/c rats after intranasal LPS treatment led to a decrease in the number of inflammatory cells in the BALF. Levels of proinflammatory cytokines, including TNF-α, IL-1, and IL-6, have also been shown to be significantly reduced in the BALF of rats after kaempferol therapy. One study has suggested that the anti-inflammatory properties of kaempferol may be associated with its ability to inhibit the activation of the mitogen-activated protein kinase (MAPK) and NF-κB signaling pathways, reducing tissue damage and pneumonia 95. Wasi *et al.* found that flavonoids protect against pinocembrin and 5,7-dihydroxyflavone in LPS-induced inflammatory reactions *in vitro* and *in vivo* 96. Dosage-dependent reduction in phosphorylation of IB, extracellular signal-regulated kinase 1/2 (ERK1/2), c-Jun N-terminal kinase (JNK), and p38 MAPK significantly inhibits the synthesis of TNF-α, IL-1, IL-6, and IL-10 *in vitro* in the presence of 0-300 g/ml pinocembrin. LPS-induced pulmonary edema and infiltration of neutrophils, lymphocytes, and macrophages were significantly reduced in rats given the anti-inflammatory drug pinocembrin at 20 or 50 mg/kg intraperitoneally. Moreover, pretreatment with pinocembrin reduced considerably levels of TNF-α, IL-1, and IL-6 but increased IL-10 levels. This study found a decrease in LPS-induced lung damage, with pinocembrin found to inhibit IB, JNK, and p38 MAPK activity (**Table 1**) 96.

Studies have examined the development of paraquat (PQ)-induced ALI and pulmonary fibrosis using naringin (Nar) as a protective agent, exploring the probability of survival after PQ poisoning at a 50 mg/kg dose in 10 km male and female rats randomly assigned to one of five groups: PQ, N-acetylcysteine (NAC), Nar1, and Nar3. The PQ group was injected intraperitoneally with PQ at 50 mg/kg. The NAC group was administered NAC intragastrically at 1 g/kg/d for three days. The Nar1 and Nar3 groups were administered naringin at 30 mg/kg/d and 120 mg/kg/d, respectively, for three days, then PQ at 50 mg/kg for three further days. The rat died of PQ poisoning after just seven days. It was found that mortality rates in the Nar1 and Nar3 groups were 20-60% of that of the PQ and NAC groups. Moreover, levels of TNF-a, TGF-β1, MMP9, TIMP metallopeptidase inhibitor 1 (TIMP1), pulmonary malonaldehyde, and pulmonary fibrosis deposition after PQ-inducement were attenuated significantly by naringin at 60 or 120 mg/kg 97.

Punicalagin can also relieve LPS-induced ARDS. Dexamethasone (5 mg/kg) and punicalagin (12.5, 25, and 50 mg/kg) were administered by intraperitoneal injection one hour before LPS (20 mg/kg) intranasal administration to induce lung injury. In addition, punicalagin has been given to the patients to minimize myeloperoxidation and neutrophil and macrophagic infiltration in the lungs. Moreover, punicalagin has been found to decrease Toll-like receptor 4 (TLR4) expression and NF-κB activation, with NF-κB activation and proinflammatory cytokine production potentially inhibited by lower *TLR4* expression 98.

## Current progress toward clinical applications

Polyphenols are bioactive chemical compounds that are found mainly in plants such as cocoa, tea, and coffee and fruits such as grapes, pomegranates, and apples and were found to be beneficial in extensive clinical studies 141-144. Polyphenols are exogenous antioxidants that play a vital role in protecting cells 145. They are a vast family of therapeutically active phytochemicals, including several that are currently being tested in preclinical and clinical studies, and have shown promising outcomes for treating respiratory disorders such as asthma, COPD, pneumonia, lung cancer, and influenza. This section illustrates some of the clinical studies on polyphenols to understand their future potential better.

### Influenza

The influenza viruses cause widespread annual epidemics and pandemics in humans and animals, resulting in significant mortality and morbidity 146. Despite the vast repertoire of pharmacotherapeutic options available for suppressing specific influenza pathogenic processes, establishing more efficient therapy options is proving challenging. A literature review by Bahramsoltani *et al.* found various natural polyphenol extracts, including pomegranate, *Cistus incanus*, lychee fruit, *Glycyrrhiza uralensis* juice, *Aronia melanocarpa*, tea, cranberry, and phenolic compounds such as quercetin, resveratrol, and caffeic acid affected influenza viral infection 147. *C. incanus* extract has favorable therapeutic benefits in modulating inflammatory biomarkers in respiratory tract diseases such as influenza 148. In a randomized, placebo-controlled study, healthcare workers who took tea catechins and theanine capsules had lower rates of influenza infection 149. Moreover, a study of 124 individuals found a significant decrease in the incidence of influenza in those taking polyphenols as adjuvant therapy 150. These clinical studies demonstrate the importance of natural polyphenols as adjuvant therapy for managing influenza with conventional drugs.

### COPD

COPD is a chronic inflammatory lung condition with deficiently reversible airflow restriction where, in most cases, the inflammatory stimulus is initiated primarily by inhalation of noxious gases, mainly CS 151. TNF-α and IL-6 are critical proinflammatory mediators that, along with matrix metalloproteinases (MMPs), are strongly associated with lung injury and healing in COPD patients. These inflammatory mediators and MMPs function interdependently. Resveratrol is a natural polyphenol found in various plants, including nuts and fruits, but is particularly prevalent in red wine and grapes. Several previous studies have shown that resveratrol has diverse anti-inflammatory properties 152-154.

A study with 34 COPD patients and 30 healthy individuals placed into four treatment groups (**Table 2**) found that nuclear translocation of NF-κB and secretion of MMP9 and TNF-α were elevated in the COPD patients compared to healthy individuals. In contrast, genistein and resveratrol inhibited the nuclear translocation of NF-κB and reduced the secretion of MMP9 and TNF-α. Therefore, these findings show that genistein and resveratrol may be promising medicinal candidates for COPD management 58.

### Lung cancer

With roughly 80% of lung cancer patients histologically categorized with NSCLC, it has become the leading cause of mortality among human malignancies. It is incurable in several cases because of resistance to many anticancer drugs 155. The therapeutic modalities available for the treatment of lung cancer include surgical intervention, chemotherapy, radiation therapy, and targeted pharmacotherapy. 156. For over two decades, combination therapy with cisplatin has been the most effective therapy for NSCLC. However, the long-term survival rate continues to be low 157. Ginger and green tea are both abundant in antineoplastic and antioxidant polyphenols. Green tea contains epicatechin (EC), epigallocatechin (EGC), EGCG, epicatechin-3-gallate (ECG), and theaflavin, which are collectively known as green tea polyphenols (GTPs) 158. Green tea and ginger, known for their health advantages, include polyphenols, plant-based chemicals with antioxidants, and other beneficial properties. Green tea mainly contains catechins, whereas ginger is rich in gingerols and shogaols, in addition to some catechins 159. Studies indicate that polyphenols in these ingredients may enhance their health advantages and perhaps have synergistic effects when ingested together 22. Ginger contains various polyphenols, including gingerol and shogaol, which have also been shown to have anti-cancer properties, including inhibiting the proliferation of lung cancer cells 160.

Catechins affect various molecular and cellular targets associated with cell death and survival, inhibiting cell proliferation and regulatory pathways associated with invasion, growth factor-related proliferation, and angiogenesis 161. A population-based study performed with 582 lung cancer patients in Hawaii 162 found an inverse relationship between lung cancer and the intake of polyphenols such as quercetin and naringin from food sources such as white grapefruit, apples, and onions, with a statistically significant 40-50% decrease in lung cancer risk in patients with high polyphenol intake compared to those with low polyphenol intake. This finding is supported by other polyphenol studies showing positive results with lung cancer 9. Phase II research was carried out to confirm the effectiveness and safety of EGCG in treating ARIE. Participants from the Shandong Cancer Hospital and Institute in China were recruited for the research. EGCG was given during the first occurrence of ARIE and weeks after the completion of radiation. The patients were observed for dysphagia, RTOG score, and discomfort associated to esophagitis. The tumor response rate was 86.3%, whereas the overall survival rates were 74.5%, 58%, and 40.5%. Administering EGCG solution orally seems to be a viable treatment for acute radiation-induced esophagitis in patients with esophageal cancer undergoing radiation therapy 163. A Chinese phase I study shown that EGCG might be a potential treatment for acute radiation-induced esophagitis (ARIE), a common side effect of thoracic irradiation. 37 patients with stage III lung cancer participated in the experiment, receiving either concurrent or sequential chemo-radiation or radiotherapy alone. EGCG was given at a dosage of 440μmol/L once Acute Radiation-Induced Esophagitis (ARIE) developed, and continued for two weeks post-radiotherapy. After administering EGCG and radiation, there was a significant reduction in RTOG score and pain ratings. The experiment verified that oral EGCG is a successful and secure approach for managing ARIE, and a phase III randomized controlled trial is anticipated to validate its efficacy 164.

### Asthma

Asthma is a severe airway condition characterized by airway inflammation, hyper-reactivity, and restricted airflow with airway remodeling 165. The development of asthma is connected with two possibly new genes, SETDB1 and ZNF8, as determined by gene-smoking interaction analysis conducted on different cohorts of the KoGES collaboration 166. Previous studies regarding the *in vivo* and *in vitro* anti-asthmatic and anti-allergic features of flavonoids strongly support the use of flavonoids as a dietary treatment or preventive strategy for asthma and other allergic human diseases 167-170. Moreover, recent clinical studies on flavonoids found that they can reverse allergic rhinitis effects 171-174. Pycnogenol is an extract of water-soluble bioflavonoids from maritime pine containing proanthocyanidins that were found to be beneficial for asthma in a randomized, placebo-controlled, double-blinded study of asthmatic individuals with different levels of severity 175.

Similarly, another study of 76 asthma patients suggested that the anti-inflammatory properties of pycnogenol could be beneficial when used in conjunction with inhaled corticosteroids (ICS), reducing ICS dosage and frequency 176. Moreover, other studies have found pycnogenol to be effective in asthma management 176,177 and collectively support the use of pycnogenol as an adjunct therapy for severe asthma. A study in a community-based trial at a single center discovered that berry fruit extract might decrease chronic airway inflammation and alter airway remodeling in models of lung inflammation generated by allergens. The trial included 28 mild asthmatics who had not used steroids before and had Feno levels of more than 40 ppb. Of them, 25 participants completed both therapies. Participants were randomly allocated to receive either 100 mg of berry fruit polyphenolic extract (BFPE) or a placebo for four weeks, with a 4-week break between the treatments. The primary variable assessed was the FeNO level after four weeks. The study found that BFPE did not have an impact on FeNO levels, which is a biomarker of eosinophilic airway inflammation, in steroid-naïve individuals with moderate asthma and high FeNO levels. It is essential to be cautious when assuming that the improvements shown in mice with lung eosinophilia would translate to effective treatment in asthma patients 178.

### Common cold

The most common infectious disease in humans is the acute upper respiratory tract virus infection, generally called the common cold. Polyphenols were effective against cold and flu viruses in some ex vivo tests with γδ-T cells 179. In addition, clinical trials have also assessed the beneficial effects of polyphenols (Gallic acid, catechin, epigallocatechin gallate, epicatechin, epicatechin gallate, and ascorbic acid) on the common cold. A randomized, placebo-controlled, double-blinded clinical study (**Table 2**) of 100 patients aged 20 to 65 with at least one cold-related local finding (e.g., throat) who scored at least 5 points based on the severity of five cold symptoms were instructed to drink 2 to 250 mL of a test or placebo beverage twice a day for ten days. Clinical examinations were scheduled prior to the start of treatment (baseline), 3-6 days later after the start of therapy (second), and 7-10 days after the start of treatment (third). By the third examination, 19 of the 49 patients in the test beverage group (38.8%) were free of symptoms compared to just 4 of the 47 patients in the placebo group (8.3%). The difference was magnified in the patient evaluations, where 41.9% of patients in the test beverage group reported being symptom-free on the evening of day 7, compared to just 5% of patients in the placebo group. This study clearly demonstrates that individuals suffering from common cold symptoms can benefit from drinking polyphenol-rich beverages, with symptoms disappearing faster than those who do not 180.

### Pneumonia

Bronchial or lobular pneumonia is a frequent infection affecting children, particularly toddlers, and newborns 181. Bronchial pneumonia in children is caused by a pathogenic bacterial, mold, or viral infection 182,183. The polyphenol naringenin has been found to have free radical scavenging, anti-atherosclerotic, anti-oxidative, anti-inflammatory, and cell protection properties and pharmacological functions such as anti-microbial and anti-cancer activity and inflammation resolution 184-187. In addition, multiple animal studies have demonstrated the usefulness of naringenin for treating respiratory diseases such as asthma and COPD 188. A clinical study with 180 patients randomly placed into one of two groups, naringenin and azithromycin (AZI; **Table 2**), assessed their blood-borne cytokine levels following a five-day oral dosing protocol and compared the time required for clinical symptoms to subside following therapy and recurrence of complications. They found naringenin to minimize the frequency of pneumonia complications and other adverse responses. It improved patient health by inhibiting inflammation, providing new insights into the potential medical applications for naringenin 189.

Polyphenol research is unquestionably a promising and exciting scientific area. However, several missing connections must be addressed regarding their efficacy for various allergic illnesses before their widespread use in disease prevention and treatment. Firstly, approaches are required to increase polyphenol bioavailability and, eventually, its beneficial effects 146,190,191. Secondly, cutting-edge biochemical techniques are required to identify active components within various polyphenol extracts to ascertain which polyphenol is associated with a given anti-allergic activity 192-194. Thirdly, the administration route (oral vs. topical) and intervention window (prevention vs. treatment) of polyphenols require further study for various allergy symptoms. Finally, the long-term estimated risk of therapeutic and dietary polyphenol use in different age groups (children, adults, and older people) must be assessed. These studies are required to confirm the general assumption that polyphenols are harmless since their content and tolerability from different extract sources can differ 195-198.

## Concluding remarks and future directions

Various polyphenol-related beneficial effects on lung disease are associated with the polyphenol content of almost all plant-based foods (fruits and vegetables). Preliminary research has shown that metabolic syndromes, such as diabetes, hypercholesterolemia, hypertension, neurodegeneration, other dementias, and various cancers, may provide new lines of preclinical research into strategies to prevent the development of various lung diseases. Nonetheless, this study focuses on the ability of EGCG, proanthocyanidin, and other biochemical compounds to treat various lung diseases. Since polyphenols are abundant in nature, it would be advantageous to study their largely unknown effects. Despite encouraging preclinical findings, only one clinical study into the efficacy of polyphenols in treating lung disease in human patients has been performed. However, it only included a small number of participants, and their follow-up period was short. The therapeutic potential of polyphenols will be better understood using larger sample sizes and longer follow-ups.

We now have a better understanding of how polyphenols are absorbed, digested, and transported throughout the body, enabling more effective clinical trials through increased knowledge about appropriate dosing. Polyphenols can be used to treat and prevent neurodegenerative conditions, where their activity results from various interconnected processes, such as their ability to scavenge free radicals and chelate metals. An additional important factor is the bioavailability of polyphenols. Multiple studies on the safety and toxicity of polyphenols have shown that high tannin levels can be harmful. Since polyphenols are present in a wide variety of foods, more research is required on their effects on humans. We believe that a proper risk-benefit analysis will accelerate the use of polyphenols as a therapy for the prevention and treatment of both acute and chronic lung disease.

## Figures and Tables

**Table 1:** Experimental evidence on the use of polyphenols against respiratory diseases.

Respiratory diseases	Polyphenolic compounds	Study model	Doses/Conc.	Results	Ref.
Asthma	Naringenin chalcone	BALB/c mice	0.8 mg/kg/day	↓ Th-2 cytokine secretion	99
	Resveratrol	Female BALB/c mice	30 mg/kg	Resveratrol lowered total IgE, OVA-specific IgE, IgG2a, IL-4, and IL-5 in plasma and BALF of OVA-induced asthmatic mice	100
	Quercetin and isoquercitrin	BALB/c mice	Isoquercitrin 15 mg/kg or quercetin 10 mg/kg	Quercetin and isoquercitrin decrease eosinophilia	101
	Kaempferol	Embryonic (BEAS-2B cells)	1-20 mmol/L	Kaempferol reduced NF-kB signaling to decrease TNF-α-induced epithelial inflammation	102
Influenza	Resveratrol	Four-week-old female BALB/c mice	1 mg/kg/day	Prevent nuclear-cytoplasmic translocation of viral ribonucleoproteins (vRNPs) and reduce the production of late viral proteins by inhibiting protein-kinase C and underlying pathways.	103
	Isoquercetin	Female BALB/c mice	female BALB/c mice	Treatment with isoquercetin dramatically decreased lung levels of IFN-, iNOS, and RANTES	104
	Baicalin	Male BALB/c mice	(10 -120 mg/kg/day	NS1 protein encoded by the IAV is modulated by baicalin to provide antiviral actions	105
	Curcumin		30 μM	Anti-infective HA activity may be used to diminish influenza virus (H1N1 and H6N1) infection.	106
Acute Respiratory Distress Syndrome (ARDS)/Acute Lung Injury (ALI)	Naringin	KM mice	30, 60, 120 mg/kg	Protects against ALI and fibrosis, suppresses oxidative stress and inflammation in the lungs, and increases antioxidant enzymes such as SOD, GSH-Px, and HO-1	97
	Pinocembrin	Male BALB/c mice	20 or 50 mg/kg, i.p.	By inhibiting p38MAPK and JNK activation, pinocembrin decreased LPS-induced lung damage	96
	Rutin, Quercetin, Icarisid II, Isorhamnetin, Chlorogenic Acid	Female C57BL/6 mice	0.15% or 0.6% *Inonotus sanghuang* (extract)ISE	An anti-inflammatory and anti-oxidation imbalance is corrected in the lungs in part because of the regulation of NF-κB signaling, which reduces inflammation.	107
	Punicalagin	Male BALB/c mice	12.5, 25, and 50 mg/kg	TLR4-mediated NF-κB signaling pathways may be a contributing factor	98
	Curcumin	Male C57BL/6 mice	intraperitoneal injection of 50 μl (20 mg/ml)	By stimulating CD4+ T cell development into FOXP3+ CD25+ Tregs, curcumin lessens the severity of ALI in mice.	94
	Kaempferol	Male BALB/c mice	25, 50 or 100 mg/kg body weight	Antioxidant and anti-inflammatory effects of kaempferol on LPS-induced ALI may be mediated via reduction of MAPKs and NF-κB signaling pathways	95
Tuberculosis	Curcumin	Tamm Horsfall Protein (THP-1) cells in a lab dish	10, 30, and 50 μM	Both apoptosis and autophagy might be mechanisms by which curcumin modulates human macrophages to increase the clearance of intracellular *M. tuberculosis*.	87
	Epigallocatechin-3-gallate	THP-1 and Jurkat cell lines		Administration of EGCG inhibits the transcription of the TACO gene in a dose-dependent manner.	108
	Silymarin	Male Wistar rats	10 mg/kg	Safely and effectively return ALT, AST, and ALP enzyme concentrations to normal.	109
COPD	Casticin	Male Wistar rats	10, 20, and 30 mg/kg)	Casticin inhibits TLR4 activation in Western blot analysis as well as the phosphorylation of NF-κB and IB.	50
	Phloretin	Male BALB/c mice	10, 20 mg/kg	It has been shown that phloretin protects against CS-related airway mucus hypersecretion and inflammation, where the EGFR, ERK, and P38 may be involved.	110
	Hesperidin	C57BL/6 mice	25, 50 mg/kg	Antioxidant stress and inflammation were decreased in CES-induced COPD model mice when the SIRT1/PGC-1a/NF-jB signaling pathway was activated.	111
	Baicalin	Rat model	20 mg/kg, 40 mg/kg, 80 mg/kg	Blocking NF-kB has an anti-inflammatory impact	55
	Oroxylin A	BEAS-2B and RAW264.7 cells	15, 30, and 60 mg/kg	The Nrf2 signaling pathway was activated by Oroxylin A to reduce CS-induced oxidative damage.	56
	Silymarin	BALB/c mouse model *in vitro* in A549 cells	20, 40 and 80 mg/Kg	Improved pulmonary function, reduced inflammation, and suppressed the production of pro-inflammatory cytokines.	112
	Fisetin	NCI-H292 and HEK293T cells	-	TRAF2 in TNF-RSC is inhibited by binding fisetin to PKC	57
	Curcumin	CS-induced rat model	100 mg/kg	Curcumin's ability to prevent alveolar epithelial damage in rats with COPD may be in part due to the downregulation of P66Shc.	113
	Naringenin	Modeling BALB/c mice using A549 cells	20, 40 and 80 mg/Kg	Naringenin administration improved lung function, decreased inflammation, and inhibited the production of inflammatory cytokines such as TNF- and MMP9.	114
Severe acute respiratory syndrome coronavirus (SARS-CoV)	Luteolin	Vero E6 cells	EC50 10 µM	The antiviral action of luteolin may be achieved by disrupting the fusion of virus cells.	115
	Emodin	Vero E6 cells	IC50 200 µM	As the dosage increased, the S protein and ACE2 interaction was significantly inhibited, as was expected.	116
	Chalcones isolated from *Angelica keiskei*	*In silico*	3CL^pro^ and PL^pro^ inhibitory activity with IC50 values of 11.4 and 1.2 mM	While the SARS-CoV PLpro demonstrated noncompetitive inhibition, the chalcones showed competitive inhibition of the SARS-CoV 3CLpro	117
	Forsythoside A	CEK cells infected with IBV	0.16 mM, 0.32 mM, and 0.64 mM	(i) dose-dependent viral load reduction, (ii) IBV nucleocapsid protein expression reduction, and (iii) dose-dependent inhibition of IBV infection	118
MERS-CoV infection	Resveratrol	Vero E6 cells infected with MERS-CoV	250-7.8125µM	(i) ↓cell death, (ii) ↓ viral RNA replication inhibition, (iii) ↓ viral titer, (iv) ↓ nucleocapsid protein expression, (v) ↓ apoptosis	119
Pulmonary fibrosis	Resveratrol, Quercetin, Mangiferin, Dihydroquercetin (DHQ)	Male CD-1 (CD1(ICR)) mice	50 mg/kg, 10 mg/kg, 10 mg/kg, 10 mg/kg respectively	1. Anti-inflammatory effects of resveratrol and quercetin 2. Protective properties of exogenous administration of mangiferin and DHQ	120
	6-Gingerol	C57BL/6 mice	100 or 250 mg/kg	6-gingerol has been shown to reduce lung fibrosis by activating SIRT	121
Pulmonary hypertension	Resveratrol	Sprague-Dawley rats	3 mg/kg	↓ pulmonary hypertension caused by MCTs	122
	Trimethoxystilbene (TMS)	Sprague-Dawely rats	5 or 10 mg/kg per day	Inhibits the NOX/VPO1 pathway, which causes oxidative stress and inflammation	123
Lung Cancer	Hesperidin	Swiss albino mice	25 mg/kg	Changing COX-2, MMP-2, and MMP-9 expressions provides anti-carcinogenic effects against lung cancer.	124
	Baicalein	Swiss Albino mice	12 mg/ kg, 50 mg/kg	Degradation is prevented in TCA cycle enzymes and electron transport chain complexes in lung cancer-bearing mice.	125
	EGCG	Mice		EGCG's anti-cancer properties are influenced by miRNA-mediated regulation, which is implicated in all of the primary elements of its action	126
	Quercetin	A110L human lung cancer cell line		↓ invasion activity by directly suppressing MMP activities and by inhibiting monocarboxylate transporter activity	127
	Peonidin-3-glucoside	*in vitro*	10-40 µM	Preventing cancer cell invasion, migration, and the production of MMPs and u-PA by cancer cells	128
	Anthocyanidins	Mouse	0.5 mg/mouse	↓ tumor growth	129
	Xanthohumol	A549 cancer cells	14-42 µM	Inducing apoptosis and cell cycle arrest	130
	Procyanidin C1	A549 cancer cells	1.25-40 µg/mL	↓ TGF-β-induced EMT	131
	Naringenin	A549 cancer cells	100 µM	↑ TRAIL-mediated apoptosis	132
	Apigenin	*in vitro*	40-160 µM	Inducing apoptosis and DNA damage	133
	Chrysin	A549 cancer cells	10 µM	Inducing apoptosis, AMPK activation, ROS	134
	Luteolin	*in vivo*	10-30 mg/kg	↓ tumor growth	135
	Quercetin	A549 cancer cells	8.4 mg/kg	↓ tumor growth	136
	Kaempferol	*in vitro*	10-50 µM	↓ TGF-β1-induced EMT and migration	82
	Genistein	H446 cancer cells	25-75 µM	The apoptosis and cell cycle arrest of cancer cells, as well as a reduction in proliferation and migration, are all achieved by using this treatment.	137
	Resveratrol	*In vitro*	0, 25, 50 and 100 µM of resveratrol for 6 h	↓ cell proliferation and apoptosis in the study subjects	78
Non-Small-Cell Lung Cancer	Resveratrol	H1703 and H1975 human NSCLC cell lines	-	↓ XRCC1 expression in NSCLC cells may increase the efficacy of etoposide treatment	75
	Curcumin	H446 human SCLC cell line	-	Curcumin enhanced apoptosis in human SCLC NCI-H446 cells through a ROS-mediated mitochondrial mechanism.	138
	Quercetin	A549 human NSCLC cell line and xenograft model	-	Lung cancer cells can be suppressed by quercetin as an aurora B inhibitor	139
	Fisetin	A549 and H1792 human NSCLC cell lines	5-20 µM	Fisetin suppressed PI3K/Akt and mTOR signaling in NSCLC cells	140

**Table 2:** Clinical studies on the use of polyphenols against respiratory diseases.

Polyphenols	Diseases	Model/Method	Outcomes	Ref.
CYSTUS052	Upper respiratory tract infection (Bacterial infection and influenza)	A randomized, placebo-controlled clinical study with 160 patients, including 56 men and 104 women aged between 7 and 81 years, suffered from an upper respiratory tract infection by medical signs. Throat Swabs were collected and placed in an appropriate bacterial culture medium to determine the infection's pathogen	129 patients finished the study out of 160 participants After four days of treatment, a significant decrease in the symptoms with inflammatory markers, including CRP and FVIII, was seen	148
Gallic acid, Catechin, Epigallocatechin gallate; Epicatechin; Epicatechin gallate	Common cold	A prospective, randomized, placebo-controlled, double-blinded, multi-centric clinical study with 100 patients (age 20 -65 years): Patients were told to intake 2 to 250 mL of the test beverage or placebo beverage twice per day for ten days	41.9 percent of patients receiving test beverages reported being complaint-free on the evening of research day 7, compared to 5 percent of patients in the placebo group	180
Quercetin	Lung cancer	A case-control study with a large population (EAGLE) was conducted in Italy. An investigation was done with 1822 incident lung cancer cases and 1991 frequency-matched controls from the Environment and Genetics in Lung cancer Etiology study.	Consumption of combination fruits and vegetables, alone fruits, and only vegetables were related to a 30, 21, and 24% decreased risk of lung cancer, respectively, in a large population-based case-control research from Northern Italy. A diet high in quercetin-rich foods was associated with a 53% decreased incidence of lung cancer. Women and men, ever smokers, showed the inverse association with quercetin-rich diets, which were highest among the heaviest smokers	9
Quercetin and Naringin	Lung cancer	Five hundred eighty-two individuals with incident lung cancer were subjected to a population-based, case-control study. Patients were diagnosed with primary lung cancer at all major medical centers of the study region between January 1, 1992, and January 1, 1997	Inverse associations between lung cancer and polyphenol food sources were shown to be statistically significant, with a 40%-50% decreased risk of lung cancer, with the patients having the highest intake of polyphenols compared with the lowest category.	162
Resveratrol and genistein	COPD	Lymphocytes were extracted for NF‑κB immunocytochemical staining and analysis of TNF‑α and MMP‑9 concentration levels from 30 healthy people and 34 COPD patients, then placed into four study groups with dexamethasone, resveratrol, and genistein.	The translocation of NF‑κB was inhibited by resveratrol and genistein, and they also reduced TNF‑α and MMP‑9 concentration levels.	58
Naringenin	Bronchial pneumonia in children	One hundred eighty patients were randomly allocated to one of two groups: naringenin (NAR) and azithromycin (AZI). During the clinical intervention, all individuals were advised to follow a five-day oral dosing protocol, and their blood cytokine levels were analyzed.	Naringenin was able to minimize the frequencies of bronchial pneumonia complications and related adverse responses, as well as enhance the health of the patients, by inhibiting inflammation and shortening the time it took for clinical signs to vanish.	189
Cranberry polyphenols	Colds and influenza	A randomized, double-blinded, placebo-controlled intervention study to see if cranberry polyphenols might affect immunity, particularly γδ -T cell proliferation. For this purpose, a total number of 54 healthy individuals, including 17 men and 37 women, aged between 21 to 50 years old and with BMI between 18 to 30 kg/m^2, were studied. 45 subjects (83%) out of 54 completed the study	After ten weeks of cranberry beverage intake, the levels of γδ-T cell proliferation were nearly five times higher, with reducing number of symptoms related to colds and influenza.	179
Catechin	Influenza	A study with 124 individuals (age limit- at least 65 years) was conducted in which 76 out of 124 subjects, including 24 men and 52 women, gargled with tea catechin extract, whereas 48 subjects gargled without tea catechin extracts and were divided into catechin group and control group consecutively.	The incidence of influenza infection decreased significantly from 1.3 percent in the catechin group to 10 percent in the control group.	150
Theanine and green Tea catechins.	Influenza	During a 5-month randomized, placebo-controlled, double-blinded experiment of 200 healthcare professionals, 98 were given green tea and theanine capsules, and 99 were given a placebo	When compared to the placebo group, the catechin/theanine group had a significantly lower incidence of both clinically and laboratory-confirmed influenza infection	149
Carvacrol	Asthma	Forty individuals with mild to severe asthma for two months	Improvements in respiratory symptoms and PFT readings were seen as a consequence of the study	199
Pomegranate juice (Ellagitannins)	COPD	30 patients for five weeks	Adding pomegranate juice to existing COPD treatment does not improve outcomes	200
EGCG, EGC, ECG, EC, and catechin gallate	Influenza infection	76 adult persons for three months	They had a reduced rate of influenza infection in the catechin-treated group compared to the control group	150
EGCG	Esophagus Cancer	51 patients	EGCG solution may be effective in treating ARIE in esophageal cancer patients receiving radiation therapy, potentially acting as an ARIE-reliever without compromising radiation therapy efficacy.	163
